# Are the sensorimotor control capabilities of the hands the factors influencing hand function in people with schizophrenia?

**DOI:** 10.1186/s12888-023-05259-w

**Published:** 2023-11-07

**Authors:** Yu-Jen Lai, Yu-Chen Lin, Chieh-Hsiang Hsu, Huai-Hsuan Tseng, Chia-Ning Lee, Pai-Chuan Huang, Hsiu-Yun Hsu, Li-Chieh Kuo

**Affiliations:** 1 Department of Occupational Therapy, College of Medicine, National Cheng Kung University, No.1, University Road, Tainan City, 701 Taiwan; 2 Department of Occupational Therapy, Da-Yeh University, Changhua, Taiwan; 3 Department of Psychiatry, National Cheng Kung University Hospital, College of Medicine, National Cheng Kung University, Tainan, Taiwan; 4 Department of Physical Medicine and Rehabilitation, National Cheng Kung University Hospital, College of Medicine, National Cheng Kung University, No.1, University Road, Tainan City, 701 Taiwan; 5 Department of Biomedical Engineering, College of Engineering, National Cheng Kung University, Tainan, Taiwan; 6 Medical Device Innovation Center, National Cheng Kung University, Tainan, Taiwan

**Keywords:** Schizophrenia, Hand function, Sensorimotor, Motor control, Assessments

## Abstract

**Background:**

Previous works reported people with schizophrenia experienced inferior hand functions which influence their daily participation and work efficiency. Sensorimotor capability is one of indispensable elements acting in a well-executed feed-forward and feedback control loop to contribute to hand performances. However, rare studies investigated contribution of sensorimotor ability to hand functions for people with schizophrenia. This study aimed to explore hand function in people with schizophrenia based on the perspective of the sensorimotor control capabilities of the hands.

**Methods:**

Twenty-seven people at the chronic stage of schizophrenia were enrolled. The following assessment tools were used: the Purdue Pegboard Test (PPT) and the VALPAR Component Work Sample-8 (VCWS 8) system for hand function; the Self-Reported Graphic version of the Personal and Social Performance (SRG-PSP) scale for functionality; and the Semmes-Weinstein Monofilaments (SWM), the pinch-holding-up-activity (PHUA) test and the Manual Tactile Test (MTT) for the sensory and sensorimotor parameters. The Clinical Global Impression-Severity (CGI-S) scale and the Extrapyramidal Symptom Rating Scale (ESRS) were used to grade the severity of the illness and the side-effects of the drugs. Spearman’s rank correlation coefficient was used to analyze associations among hand function, functionality, and sensorimotor capabilities. A multiple linear regression analysis was used to identify the determinants of hand function.

**Results:**

The results indicated that both hand function and sensorimotor capability were worse in people with schizophrenia than in healthy people, with the exception of the sensory threshold measured with the SWM. Moreover, the sensorimotor abilities of the hands were associated with hand function. The results of the regression analysis showed that the MTT measure of stereognosis was a determinant of the PPT measure of the dominant hand function and of the performance on the VCWS 8, and that the ESRS and the MTT measure of barognosis were determinants of the performance on the assembly task of the PPT.

**Conclusions:**

The findings suggested that sensorimotor capabilities, especially stereognosis and barognosis, are crucial determinants of hand function in people with schizophrenia. The results also revealed that the side effects of drugs and the duration of the illness directly affect hand function.

**Clinical Trail Registration:**

ClinicalTrials.gov, identifier NCT04941677, 28/06/2021.

## Background

Schizophrenia is a lifelong disease that adversely impacts an individual’s ability to perform daily routines and occupational duties. In addition to its well-known positive and negative symptoms such as hallucinations, delusions or socially withdrawn, motor impairment in schizophrenia has also been found to interfere with the ability to accomplish activities of daily living (ADL) and with work efficiency [1]. Motor function deficits, especially hand motor impairments, affect the performance of ADL, the quality of social interactions, and work participation [2–4]. The major rehabilitation goal for individuals at the stable or chronic phase of schizophrenia is being able to successfully lead an independent life [5]. Unfortunately, impairments in hand function such as poor finger dexterity, psychomotor slowness, tremors, and inadequate finger force control have been observed in people with stable schizophrenia [1, 6], potentially affecting their performance on both everyday tasks and work-related tasks. In contrast, better hand function in people with schizophrenia has been found to positively correlate not only with the number of days they were able to work, their work-related income and their vocational outcomes, but also with social interaction [2, 7, 8].

According to motor control theories, hand function efficiency relies heavily on well-integrated feed-forward and feedback control mechanisms [9]. When a person initiates a movement, the motor plan is recalled by a feed-forward control mechanism, which prompts the relevant muscles to produce the appropriate motor output. The occurrence of sensory prediction errors as the movement progresses results in online motor corrections in order to achieve the precise motor output [9]. Therefore, sensory, motor, and sensorimotor capabilities are elements of a properly functioning feed-forward/feedback control loop which are indispensable to effective hand performance [10]. More specifically, sensorimotor integration plays a significant role in impairments of the upper extremities in patients with psychiatric disorders [11]. The conventional clinical approach involves measuring the sensory threshold, range of motion, grip strength, and manipulation skills of patients in order to evaluate upper extremity impairment [12]. However, the tests that are currently used to perform these measurements seem to be incapable of identifying sensorimotor deficits in the upper extremities. The Manual Tactile Test (MTT) and the pinch-holding-up-activity (PHUA) test are recently developed, easy-to-use methods which are extremely effective at helping clinicians to assess sensorimotor integration and to predict sensorimotor performance in the hands [13]. The MTT is a tool which makes use of manual exploration to assess discriminative sensations in response to differences in the weight, texture, and shape of various objects [14]. The PHUA, on the other hand, is a valid test that examines online motor corrections of sensory prediction errors by evaluating the spatial and temporal parameters of pinch force control during a pinch-lifting movement [15]. However, there is a lack of research justifying the use of these two tests for evaluating sensorimotor integration in patients with schizophrenia.

The sensory function of individuals with schizophrenia has also been discussed. G Zengin, MR Yzzici and H Meral [16] examined an eight-week sensory-based occupational therapy program for individuals with early-onset schizophrenia. Their results showed that implementing a developmental occupational therapy program incorporating sensory integration-based techniques improves sensory processing skills, positive and negative symptomatology, cognitive symptoms, and creative abilities for individuals with schizophrenia. To better understand the sensory processing patterns in individuals with schizophrenia, G Zengin and M Huri [17] explored the relationship between sensory processing patterns, substance use, and schizophrenia. They found differences in sensory patterns between those with schizophrenia and substance use disorders and those with schizophrenia only and a correlation between sensory patterns of individuals with schizophrenia with substance use and positive symptoms.

A recent study reported that some people with schizophrenia exhibit a variety of motor abnormalities that might be due to dysfunctional feed-forward and feedback mechanisms but not to a deficit in higher cognitive functions [18]. Even if the feed-forward mechanism has been found to be partially retained in schizophrenic patients [19], it has been suggested that impaired timing of the cortical response to tactile stimulation [20] and deficits in the processing of somatosensory information [21] cause these patients to incorrectly predict the sensory inputs resulting from their actions, resulting in ineffective motor control [6, 10, 22–24]. Impaired hand function is known to have a deleterious effect on work performance, participation in daily life and quality of life for various populations, including people with schizophrenia. Notwithstanding the efforts that have been made in studies such as these to understand and treat hand function impairments in people with schizophrenia, much work remains to be done. In particular, comprehensive assessments should be conducted in order to simultaneously examine the roles of various neglected factors of the sensory or sensorimotor integration of hand capabilities.

Hand function in people with schizophrenia has been found to negatively correlate with their negative symptoms, but it did not significantly correlate with demographic features such as age, gender, and education level [22]. However, there is still insufficient evidence to fully comprehend the relationship between sensorimotor parameters and hand function and to explore the effect of such determinants as demographic characteristics, disease features and drug effects which may be related to hand function in patients with schizophrenia.

Therefore, the first aim of this study was to quantify impairments of the motor, sensory, and sensorimotor integration of the hand capabilities in people with schizophrenia. The measurements would then be used in an attempt to explore the relationships among the sensory parameters, the motor parameters, the sensorimotor parameters, hand function, and functionality in these people. The second aim was to clarify the determinants of hand function in relation to schizophrenia, e.g., investigating the sensorimotor parameters with demographic variables and clinical characteristics. We hypothesized that sensorimotor parameters (1) correlate with hand function and functionality and (2) act as predictors of hand function in patients with schizophrenia.

## Methods

### Study design and ethical statement

This is a cross-sectional, observational study which investigates hand function and corresponding factors in participants with chronic schizophrenia. The study protocol was approved by the Institutional Review Board of the National Cheng Kung University Hospital (B-ER-109-188). Prior to taking part in this experiment, each participant was informed of its purposes and the procedures that would be followed. They then gave their written informed consent to participate in the study.

### Participants

People with schizophrenia who were registered in a local rehabilitation program were referred to us by psychiatrists at the National Cheng Kung University Hospital between September 2020 and June 2021. The criteria for determining the appropriate sample size (n = 29) were based on a 2-tailed alpha of 0.05, a statistical power of 0.8 and a large effect size (q = 0.5) [25]. The inclusion criteria were: (1) A diagnosis of schizophrenia or schizoaffective disorder by a psychiatrist using the DSM-5 [26]; (2) being aged 20–65 years old; (3) taking the same antipsychotics and exhibiting stable symptoms for at least one month before the study; and (4) being able to follow instructions and having adequate cognitive abilities, which corresponds to a score on the Mini-Mental State Examination (MMSE) of higher than 24 [27]. Applied to the same one-month period prior to the study, the exclusion criteria were: (1) Substance addiction and other medical conditions (such as disease-related peripheral neuropathy) that may affect sensorimotor or movement functions; and (2) using non-antipsychotic medications that may affect movement.

### Instruments

#### Questionnaires on subject data

Basic data on the research subjects was collected by means of a self-report questionnaire and a chart review questionnaire. The demographic and disease-related information that was gathered included gender, current age, age at first episode, duration of illness after diagnosis, education level, occupational status, use of antipsychotic or anticholinergic medication, outpatient/inpatient status, and dominant/nondominant hand. It took about five minutes to complete the questionnaires.

#### Semmes-Weinstein Monofilaments (SWM)

The SWM is the most responsive tool for detecting cutaneous pressure thresholds, with acceptable to good levels of reliability and specificity [28, 29]. The subjects were instructed to close both eyes, and then the filaments were oriented perpendicular to the target area and used to apply pressure to the finger pulp with a constant force for 1-1.5 s. Meanwhile, the subjects were asked to respond by saying the word “touched” when they felt the monofilament stimulating their skin. A score was recorded based on a numerical rating assigned to each monofilament corresponding to the logarithm to the base ten of the force measured in tenths of a milligram. Higher SWM scores indicated poorer sensory function. The target force (in grams) of the thinnest filament that was felt by the subject was recorded as the result.

#### Pinch-holding-up-activity (PHUA) test

**Fig. 1:**
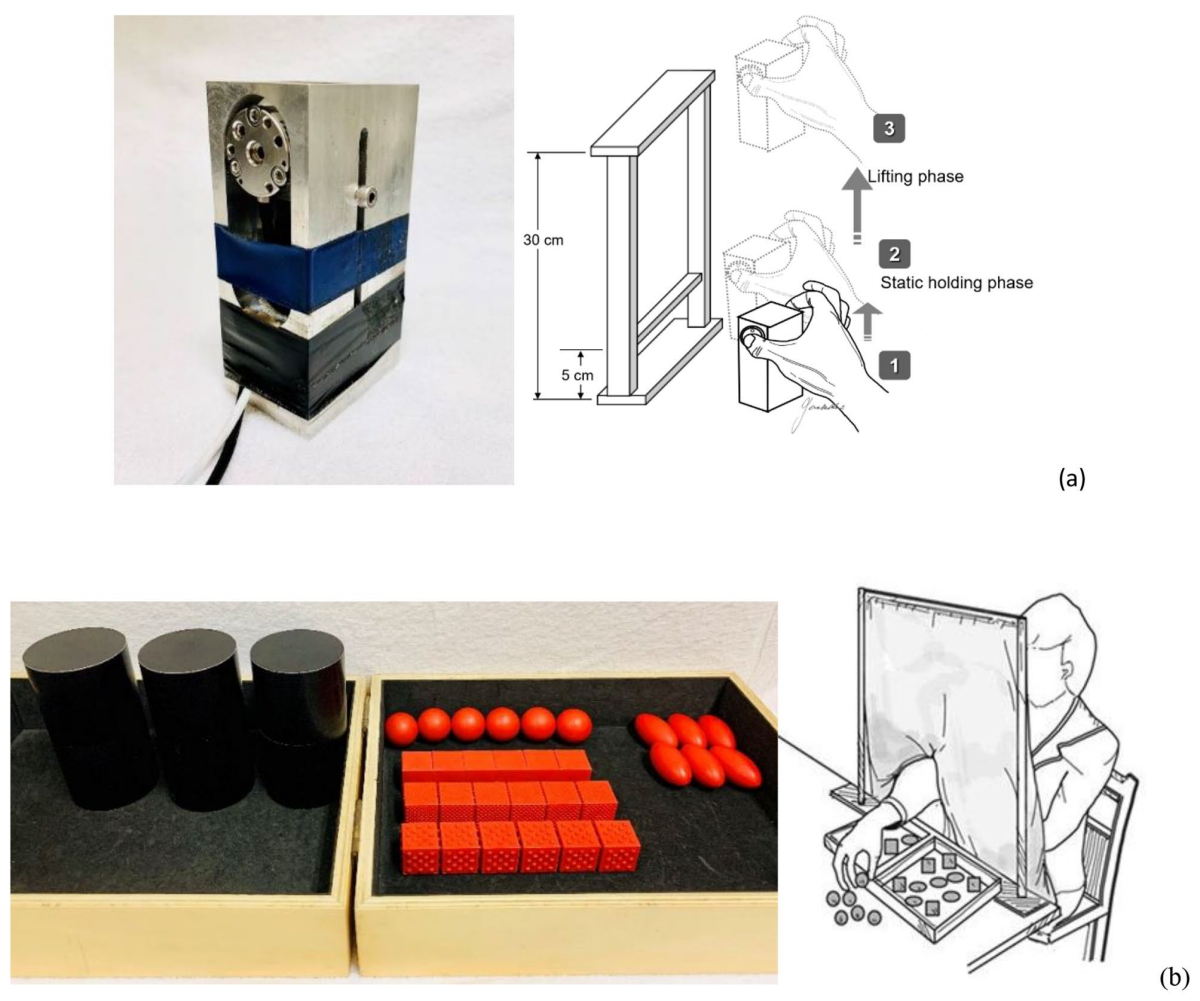
(**a**) The pinch apparatus and testing procedures of the PHUA assessment; and (**b**) the subtests of the MTT and testing scenario

The PHUA test is a task-based assessment tool with high reliability which was designed to quantify the sensorimotor capabilities of the hand by detecting pinch force modulation capability in response to movement-induced load force fluctuations [30]. The apparatus used to measure the pinch force was a steel square cuboid (6 × 3.65 × 3.65 cm) with a weight of 480 g (Fig. 1a). Embedded in the apparatus, two 6-axis load cells and one triaxial analog accelerometer were used for measuring the pinch force exerted by the hand and the acceleration of the apparatus, respectively, during the testing procedure. The subjects were instructed to pinch and lift the apparatus to about 5 cm above the table with the thumb and index finger and hold it there for 5 s to establish the baseline pinch force for each subject. They were then instructed to lift the device to about 30 cm above the table and finally to lower their hand slowly. The duration of each trial was 15 s. The testing procedure was divided into two phases, a lifting phase (lifting the apparatus from 5 to 30 cm above the table) and a holding phase (holding the apparatus at the height of 30 cm above the table). Each trial was performed first with the dominant hand and then with the nondominant hand. After completing the PHUA test, the participants were asked to generate a maximal voluntary contraction with a palmar pinch, which was defined as their maximal pinch force. The parameters for the PHUA test included: (1) FP_peak_: the peak pinch force during the lifting phase; (2) FL_max_: the maximum load force at the onset of the maximum rate of upward acceleration; (3) force ratio: the ratio between the FP_peak_ and the FL_max_; and (4) percentage of maximal pinch force: the FP_peak_ divided by the FL_max_.

#### Manual tactile test (MTT)

The MTT (Fig. 1b) evaluates the ability to detect sensory information in the form of proprioceptive and tactile inputs. Subjects engage in active exploration as a way of assessing their discriminative sensations, which the MTT achieves by separately testing three distinct hand skills: barognosis, stereognosis, and roughness differentiation [14]. In the current study, each of these subtests of the MTT was performed with both the dominant hand and the nondominant hand three times each. The total time required was approximately 20 min. The average time spent to do each subtest was used as the final score of that test. The lower the score, the better the discriminative sensations of the subject.

#### Purdue Pegboard Test (PPT)

The PPT is a test of manipulative dexterity – i.e., unimanual and bimanual hand dexterity – that has shown high levels of reliability and validity [31]. The test-retest reliability of its five subtests for patients with schizophrenia has been assessed as moderate to good (ICC = 0.73–0.88) [8]. The PPT is performed by inserting pins into small holes with the dominant hand only and with both hands simultaneously as fast as possible for a duration of 30 s. It also requires that the subjects assemble as many configurations as possible of one pin, one collar and two washers in one minute. The number of pins and completed assemblies are recorded as the scores. The higher the score, the better the finger dexterity of the subject.

#### The VALPAR Component Work Sample-8 (VCWS 8) system

The VCWS 8 system (VALPAR International Corporation, Tucson, Arizona, U.S.A.) simulates light work and is used to evaluate manual dexterity and motor coordination [32]. The wheel of the VCWS 8 turns counterclockwise at a constant speed, and the participants are tasked with completing as many three-part assemblies (one pin and two spacers) in 20 min. In the current study, the number of correct assemblies was counted by means of a video recording of the task made using a digital camera.

#### Self-reported Graphic version of the Personal and Social Performance (SRG-PSP) scale

The SRG-PSP scale is a self-report scale examining the four domains of the PSP scale: personal and social relationships, socially useful activities, self-care, and disturbing and aggressive behavior. It was designed to assess functionality in psychiatric patients [33]. The overall score is the sum of the scores for all four domains. It has shown an acceptable level of internal consistency ( α = 0.79) [33].

#### Clinical global impression-severity (CGI-S)

The CGI-S scale is used to assess the symptoms, behavior, and global functioning of patients with mental disorders. It is a seven-point scale that is rated by confirming the presence of relevant symptoms and their influence on patients’ functional performance in the context of aspects of their lives such as home, school and work, and their relationships [34]. The CGI-S has previously been found to correlate strongly with both the Global Assessment of Functioning (GAF) scale (*r* = -.893, *p* < .0001) and the Positive and Negative Syndrome Scale (PANSS) for people with schizophrenia (*r* = .692, *p* < .0001) [35].

#### Extrapyramidal Symptom Rating Scale (ESRS)

The ESRS was developed to assess drug-induced extrapyramidal symptoms, including Parkinsonism, akathisia, dystonia, and tardive dyskinesia. It has shown good reliability [36]. The patients are said to have residual extrapyramidal symptoms if the results of the ESRS include either at least one item that is scored at 3 points or more, or at least two items scored at 2 points or more.

### Experimental procedure

After ethical approval was obtained for this study, the participants were recruited from the Department of Psychiatry at a medical center in southern Taiwan. The assessment process was divided into two stages. First, the PPT, VCWS 8, SWM, PHUA, and MTT took an occupational therapist around 60 min to administer. Second, residual symptoms and extrapyramidal symptoms were evaluated by means of the CGI-S, ESRS, and SRG-PSP, which were administered by an experienced psychiatrist in approximately 20 min.

### Statistical analysis

The statistical analyses were conducted with the help of the SPSS 17.0 software. The descriptive statistics, expressed as means and standard deviations, summarize the demographic features and factors regarding the assessment of motor, sensory and sensorimotor capabilities based on the parameters of the PPT, VCWS 8, SWM, and PHUA, and on the time required to complete the MTT. Spearman’s correlation coefficient was used to analyze the associations between the results of the hand function tests such as the PPT and the VCWS 8, and the measurements of the sensory, motor and sensorimotor parameters obtained with the SWM, the PHUA and the MTT. In addition, Spearman’s rank correlation was used to examine the associations among the sensorimotor parameters, clinical characteristics, hand function, and functionality. Multiple linear regression was used to identify the determinants which predicted hand function. Variables were included in the regression model based on the results of the correlation analyses. The level of significance was set at *p* ≤ .05.

## Results

Twenty-nine participants with schizophrenia were enrolled in this study. Two cases were unable to complete all tests accurately and were therefore excluded. The final number of participants included in the analysis was 27. The demographic data and the results for the sensorimotor performance, the hand function, the functional performance, the severity of the symptoms, and the residual extrapyramidal symptoms are presented in Table 1.

**Table 1:** Results of demographic characteristics, sensorimotor parameters, hand function, functionality and symptoms/ EPS residue of participants with schizophrenia

Demographic characteristics		Sensory, motor and sensorimotor parameters		Hand function/functional performance		Symptoms/ EPS residue	
Age (years)	42.6 (7.60)	**Sensory**		**Hand function**		CGI	3.25 (0.65)
Gender (Male/Female)	12/15	SWM		PPT		ESRS	5.66 (7.98)
Onset duration (years)	20.9 (7.81)	Thumb (mg)	0.08 (0.17)	Dominant test (unit)	14.74 (2.75)		
		Index finger (mg)	0.17 (0.32)	Assembly test (unit)	34.34 (9.43)		
Education level		Little finger (mg)	0.13 (0.20)	VCWS 8	199.77 (44.91)		
Elementary school	0						
Junior high school	2	**Motor**					
Senior high school	9	PHUA		**Functionality**			
College and above	16	Maximal pinch force (N)	9.40 (2.82)	SRGPSP			
Working status		**Sensorimotor**		Personal/social relationships	11.22 (3.68)		
No	7	PHUA		Socially useful activities	11.96 (3.89)		
Sheltered employment	9	Force ratio	3.16 (0.84)	Self-care	17.55 (1.39)		
Supported employment	11	% of maximal pinch force	0.42 (0.17)	Disturbing/aggressive behavior	5.92 (1.49)		
		MTT		Total score	34.81 (5.78)		
		Barognosis test					
		Dominant hand (s)	3.26 (0.75)				
		Nondominant hand (s)	3.43 (0.75)				
		Roughness differential test					
		Dominant hand (s)	35.55 (8.49)				
		Nondominant hand (s)	39.88 (10.14)				
		Stereognosis test					
		Dominant hand (s)	34.69 (7.94)				
		Nondominant hand (s)	36.41 (8.17)				

Note—Values are presented as Mean (SD)

SWM, Semmes-Weinstein Monofilaments; Radial side, DIP of index finger with palmar side; Ulnar side, DIP of little finger with palmar side; PHUA, Pinch-holding-up-activity; MTT, Manual Tactile Test., PPT, Purdue Pegboard test; SRGPSP, Self-reported version of the graphic Personal and Social Performance, CGI, Clinical Global Impression-Severity; ESRS, Extrapyramidal Symptom Rating Scale

The correlations between the sensory, motor, and sensorimotor parameters and the measurements of hand function are shown in Table 2. Our results indicated that the scores on all the subtests of the MTT correlated significantly with the PPT scores for the dominant hand (*rho* = − 0.66~-0.53, *p* < .01). In the case of the PPT scores for the assembly task, the results were similar (*rho* = − 0.56~-0.40, *p* < .05), except for the components of the MMT that evaluate barognosis of the non-dominant hand (*rho* = − 0.36, *p* = .06) and roughness differentiation (*rho* = − 0.28, *p* = .16). The force ratio determined by means of the PHUA test (*rho* = − 0.47, *p* = .001) and all the scores on the MTT subtests (*rho* = − 0.58~-0.43, *p* < .05) showed significant negative correlations with the scores on the VCWS 8, except for the scores on the roughness differentiation task performed with the nondominant hand (*rho* = − 0.37, *p* = .06). The correlations between the subjective reports of functionality and both the sensorimotor parameters and the clinical characteristics are shown in Tables 3 and 4, respectively. The total score on the SRG-PSP scale did not show a statistically significant correlation with the sensorimotor parameters, but it significantly correlated with the demographic variables of age and duration of illness (Table 4).

**Table 2:** Correlation analyses between sensory, motor, sensorimotor parameters and hand function (PPT, and VCWS 8)

	PPT				VCWS 8	
	Dominant		Assembly			
	*rho*	*p value*	*rho*	*p value*	*rho*	*p* value
**Sensory**						
SWM						
Thumb	0.15	0.45	0.07	0.72	0.09	0.66
Index finger	-0.31	0.12	-0.35	0.07	-0.11	0.59
Little finger	-0.13	0.51	-0.29	0.13	-0.08	0.71
**Motor**						
PHUA						
Maximal pinch force	-0.32	0.10	-0.19	0.34	-0.31	0.12
**Sensorimotor**						
PHUA						
Force ratio	-0.22	0.26	-0.31	0.12	-0.47	0.01*
Percentage of maximal pinch force	0.13	0.51	0.31	0.88	0.11	0.58
MTT						
Barognosis test						
Dominant	-0.63	0.00**	-0.49	0.01*	-0.43	0.03*
Nondominant	-0.53	0.00**	-0.36	0.06	-0.49	0.01**
Roughness differential test						
Dominant	-0.59	0.00**	-0.40	0.04*	-0.48	0.01*
Nondominant	-0.58	0.00**	-0.28	0.16	-0.37	0.06
Stereognosis test						
Dominant	-0.65	0.00**	-0.56	0.00**	-0.58	0.00**
Nondominant	-0.66	0.00**	-0.43	0.02*	-0.43	0.02*

Note: SWM, Semmes Weinstein Monofilaments; Radial side, DIP of index finger with palmar side; Ulnar side, DIP of little finger with palmar side; PHUA, Pinch-holding-up-activity; MTT, Manual Tactile Test

* indicates statistical significance (*p* < .05)

**Table 3:** Correlation results between sensorimotor parameters and functionality

	SRGPSP									
	Domain1		Domain2		Domain3		Domain4		Total	
	*rho*	*p* value	*rho*	*p* value	*rho*	*p* value	*rho*	*p* value	*rho*	*p* value
**Sensory**										
SWM										
Thumb	0.13	0.51	0.15	0.47	0.17	0.39	-0.12	0.55	0.24	0.23
Index finger	0.09	0.64	0.33	0.10	-0.15	0.45	0.04	0.86	0.23	0.25
Little finger	0.12	0.57	0.08	0.71	0.36	0.07	0.09	0.65	0.19	0.34
**Motor**										
PHUA										
Maximal pinch force	-0.01	0.95	0.13	0.51	-0.30	0.12	0.15	0.47	0.02	0.91
**Sensorimotor**										
PHUA										
Force ratio	-0.36	0.07	0.45	0.02*	-0.02	0.93	0.26	0.19	0.02	0.92
Percentage of maximal pinch force	-0.08	0.71	0.06	0.77	0.24	0.23	-0.51	0.80	0.33	0.87
MTT										
Barognosis test										
Dominant	0.06	0.76	-0.05	0.81	0.32	0.04*	0.15	0.47	0.07	0.72
Nondominant	-0.11	0.59	0.06	0.78	0.17	0.41	0.12	0.55	-0.02	0.93
Roughness differential test										
Dominant	-0.19	0.35	-0.01	0.95	-0.01	0.96	0.12	0.54	-0.11	0.59
Nondominant	-0.30	0.14	-0.14	0.47	0.07	0.74	0.19	0.35	-0.26	0.19
Stereognosis test										
Dominant	-0.26	0.19	0.02	0.93	0.27	0.17	0.14	0.47	-0.11	0.58
Nondominant	-0.25	0.22	-0.12	0.55	0.22	0.27	0.23	0.24	-0.20	0.33

Note: Domain 1, personal and social relationships; Domain 2, socially useful activities; Domain 3, self-care; Domain 4, disturbing and aggressive behavior; Total, total score

* indicates statistical significance (*p* < .05)

**Table 4:** Correlation results between clinical characteristics and functionality

	SRGPSP									
	Domain 1	*p*	Domain 2	*p*	Domain 3	*p*	Domain 4	*p*	Total	*p*
Age	0.28	0.16	0.51	0.01**	0.18	0.37	-0.01	0.97	0.57	0.00**
Duration	0.27	0.18	0.42	0.03*	0.30	0.12	0.19	0.36	0.49	0.01*
CGI	0.11	0.58	0.02	0.94	-0.15	0.46	0.07	0.71	0.00	0.99
ESRS	-0.13	0.53	0.28	0.16	-0.11	0.59	0.04	0.86	0.10	0.64

Note: Domain 1, personal and social relationships; Domain 2, socially useful activities; Domain 3, self-care; Domain 4, disturbing and aggressive behavior; Total, total score; p, p value; Du, onset duration; CGI, Clinical Global Impression-Severity; ESRS, Extrapyramidal Symptom Rating Scale

* indicates statistical significance (*p* < .05)

### The predictors of hand function and functional performance

The predictors of all measures of hand function based on the multiple linear regression analysis are presented in Table 5. The independent variables were the demographic data, the sensory, motor and sensorimotor parameters, and the clinical features. The stereognosis subtest in the MTT and the touch-pressure threshold of the thumb and index finger accounted for 57% of the variance of the dominant hand function as assessed by the PPT (*p* < .05). The ESRS and the barognosis subtest in the MTT accounted for 51% of the variance of the assembly hand function as assessed by the PPT (*p* < .05). Finally, the stereognosis subtest in the MTT and the illness duration variable accounted for 45% of the variance of the VCWS 8 results (*p* < .05).

**Table 5:** Multiple linear regression analysis between sensorimotor parameters and hand function

Model	Predictor	B^a^	β^b^	*p*
**PPT Dominant** F (2, 26) = 18.242, *p* < .01, Adjusted R^2^ = 0.570	Constant	22.55		
	MTT Stereognosis	-0.24	-0.69	< 0.01**
	SWM radial	-3.10	-0.37	< 0.01**
**PPT Assembly** F (2, 26) = 14.865, *p* < .01, Adjusted R^2^ = 0.516	Constant	52.59		
	ESRS	-0.75	-0.63	< 0.01**
	MTT Barognosis	-4.30	-0.35	0.02*
**VCWS 8** F (2, 26) = 11.670, *p* < .05, Adjusted R^2^ = 0.451	Constant	321.49		
	MTT Stereognosis	-3.23	-0.57	< 0.01**
	Duration	-1.90	-0.33	0.03*

Note: PPT, Purdue Pegboard test; Dominant, dominant hand subtest; Assembly, assembly subtest; MTT, Manual Tactile Test; D, dominant hand; SWM, Semmes Weinstein Monofilaments; Radial side, DIP of index finger with palmar side; bar,; Duration, illness duration; Ba, the nonstandardized coefficient; βb, standardized coefficient

* indicates statistical significance (*p* < .05)

## Discussion

The main findings of this study are that the sensorimotor parameters of people with schizophrenia not only significantly correlated with hand function, but also acted as predictors of hand function. In addition, the results of the ESRS suggest that extrapyramidal symptoms significantly influence hand function, in particular when both hands are being used simultaneously. These findings partially support our hypothesis.

### Relationship between sensorimotor parameters and hand function

The sensorimotor capabilities assessed by the PHUA force ratio and the MTT are the parameters that were most relevant to hand function. The hand functions assessed by means of the PPT and the VCWS 8 all correlated with the MTT for the dominant hand. In addition, the VCWS 8 results significantly correlated with the PHUA force ratio. While performing the lifting task of the PHUA test, subjects are required to adjust their pinch force in proportion to increases in the load force resulting from the effects of gravity and acceleration [30]. It has been shown that increases in the force ratio correspond to decreases of the same degree in the efficiency of the motor adaptation of the upper limb [37], which leads to impaired upper limb coordination and dexterity in people with schizophrenia. Moreover, the MTT consists of a 2-stage process, with a general pinching task and a more precise tactile stroking task aimed at discriminating the weight, shape, and texture of various objects as a means of objectively assessing the sensorimotor functions of the hand [14]. It has been suggested that the improvement of the accuracy and speed with which the properties of objects are perceived may lead to more efficient motor control of the hand [14], which is supported by the results of the current study. Our results are also consistent with those of another study indicating that sensorimotor integration is related to hand dexterity [6].

On the other hand, neither the motor parameters nor the sensory parameters associated with the maximum static pinch force and the touch-pressure sensory threshold significantly correlated with hand function in the current study. Similar results have been obtained in a past study with young adults in India where no significant correlation was found between grip strength, measured with a pinch meter, and hand function, assessed by means of the Nine-Hole Peg Test (9-HPT) [38]. In that previous study, it was suggested that hand dexterity requires the ability to achieve skilled, rapid and well-coordinated control rather than maximal hand strength. In another study, however, the sensory threshold, assessed in healthy adults by using the SWM, was found to correlate with hand dexterity, assessed by means of both the Jebsen-Taylor Hand Function Test (JTHFT) and the Functional Dexterity Test (FDT) [29, 39].These results seem to be inconsistent with the results of the current study on people with schizophrenia. In fact, the SWM scores of our participants did not reveal obvious deficits in their touch-pressure threshold. As hand function requires complex interactions between the central feed-forward mechanism and the peripheral sensory feedback mechanism, the hand function deficits observed in the people with schizophrenia may be partially affected by a dysfunctional feed-forward motor control mechanism [40].

### The predictors of hand function

The findings of the current study show that the predictors of hand function for people with schizophrenia included the tests for stereognosis and barognosis, the ESRS, the sensory threshold, and the duration of the illness. The stereognosis test was found to be the principal predictor. This is consistent with previous findings of a relationship between results for the Manual Form Perception Test (a test of stereognosis) and for performance measures of hand function (e.g., the JTHFT and the PPT) in the context of research on cerebral palsy [41, 42]. Accordingly, clinicians should take sensorimotor capabilities into account while assessing or training hand function in people with schizophrenia.

Regarding the symptoms of schizophrenia, the longer the illness lasts, the worse the hand function becomes. The results of the current study revealed that the presence of extrapyramidal symptoms and the duration of the illness are predictors of hand function in people with schizophrenia. Motor dysfunctions induced by the side effects of drugs have been found to be more common at the chronic stage of schizophrenia [43]. More specifically, it has been suggested that extrapyramidal symptoms are responsible for the cognitive planning involved in movement disorders associated with psychiatric disorders [44], resulting in deficits in bi-manual coordination. These results support the findings of the current study.

### Sensory and motor parameters in schizophrenia

The maximum static pinch force measured with the PHUA test, which represented motor capability in this study, was slightly lower than the average for healthy subjects [14]. This finding is consistent with that of another study investigating the power grip of people with schizophrenia by means of the Jamar hand dynamometer [45]. These results may be attributable to the fact that people with schizophrenia tend to have few opportunities to participate in various activities due to their illness and symptoms. In fact, it has been suggested that the relatively low level of activity and limited social participation of these people [3, 42, 46, 47] reduce their chances to exercise their extremities and to strengthen their grip and pinch function [48].

With regard to sensory function, our findings of poor light touch sensation in the hands were supported by the results of untypical registration level and sensory sensitivity of an individual with schizophrenia [16], the findings of a correlation study between sensory processing patterns with positive symptoms [17], and the recent findings of subtle variations in tactile acuity in people with schizophrenia [49]. A subtle reduction in sensitivity in the hands of these people may be related to impaired gating in the secondary somatosensory cortex [50]. However, to eliminate any uncertainty about the accuracy of the SWM measurements, we performed the “area-localization” procedure [51] after conducting the standard SWM assessment. Unfortunately, half of the participants did not correctly localize the stimuli. The results of this combined assessment procedure are consistent with those of a previous study examining the performance on finger localization tasks of people with schizophrenia who exhibited worse accuracy than the healthy control group [52]. However, the topographic analysis of sensitivity performed in the current study revealed comparatively excellent discriminatory ability, given that the degree of sensitivity required for subjects to perceive the thinnest SWM filament is much higher than the sensitivity that can be measured with the localization test. Although we did not take into account the influence of the cognitive impairments of the participants, we have to admit that we doubt the credibility of the findings of such sensory assessments conducted with people with schizophrenia. Our observations during the process of evaluating the participants’ performance have led us to believe that the impact of the illusions and disorganized symptoms encountered in schizophrenia are not negligible when fine sensory thresholds are measured with standard assessment tools. Therefore, it is crucial for researchers interested in evaluating sensitivity in people with schizophrenia that a suitable sensory assessment instrument should be developed.

The strength of this study is the finding that the MTT and the PHUA test are effective tools for assessing the sensorimotor capabilities of people with schizophrenia. As far as we know, few studies have been conducted to assess these abilities. We obtained a strong correlation between the sensorimotor parameters, evaluated by means of the MTT and PHUA test, and hand function. Moreover, the sensorimotor parameters proved to be strong predictors of bilateral upper limb coordination. Our participants produced a maximal pinch force, assessed with the PHUA test, of 42% compared to approximately 25–30% by healthy control subjects in previous studies [37, 53], and our subjects exhibited a force ratio of 3.16 which is higher than the value of 2.5 previously obtained with healthy subjects [14, 53]. These results indicate that schizophrenia is associated with poor sensorimotor control of the hands. Furthermore, the results are similar to those of a recent investigation of the sensorimotor control of the upper extremities for people with schizophrenia using a force-tracking test [6]. Regarding the MTT, on the other hand, the time required by our subjects to perform all the tasks was longer than the time needed by previously evaluated healthy subjects [14]. As far as we know, ours is the first study to measure the sensorimotor capabilities of the hands of people with schizophrenia by means of these two sensorimotor assessment tools. The deficits in sensorimotor control exhibited by people with schizophrenia may be explained on the basis of the previous finding in neuroscience research (using transcranial magnetic stimulation and functional magnetic resonance imaging) that an imbalance between excitability and inhibition in the primary motor cortex may cause sensorimotor impairments in people with schizophrenia [10, 54].

### Limitations and future work

This study only examined people with chronic schizophrenia who had taken part in a community rehabilitation program, but we did not consider data on hand function and sensorimotor performance for people at the acute and subacute stages of schizophrenia. To be able to create a more comprehensive picture of hand function and sensorimotor performance in people with schizophrenia, it is imperative to collect data on people at various stages of the illness and on a wider range of subject characteristics.

A second limitation lies in the fact that the SWM, our instrument of choice for evaluating the tactile detection threshold of the hand, proved to lack in sensitivity and accuracy in our investigation of hand function with a schizophrenia population. Although it is well known that the SWM test is commonly used for evaluating hand sensitivity in clinical settings, it seems that it was not suitable for use with our cohort of people with schizophrenia. In addition, the small sample size is a limitation that cannot be disregarded. Two participants were unable to fulfill all the tests correctly.

Although we were initially committed to producing a comprehensive picture of hand function in people with schizophrenia, our failure to take into consideration findings from brain-imaging research means that we were not able to examine the role of the activation of the motor cortex in sensorimotor processing. Future work on sensorimotor capability might explore the role of brain activation by means of functional magnetic resonance imaging or near-infrared spectroscopy. Moreover, as the effectiveness of hybrid neurorehabilitation has been confirmed [55, 56], the treatment effects of integrated sensorimotor approach into traditional psychoeducation and psychotherapy on functional performances of individuals with schizophrenia should be investigated in the near future. Finally, future studies on schizophrenia could simultaneously investigate all drugs taken by the subjects in order to further explore the effects of different drugs on sensorimotor parameters.

## Conclusions

In summary, this study of people with schizophrenia provides findings from various assessments of hand function from the perspective of various sensorimotor parameters, and it clarifies the determinants of hand function based on the feedback control mechanism. Thus, our understanding of hand function is enhanced. The findings show that hand function and sensorimotor performance were worse in people with schizophrenia compared with healthy research subjects. Moreover, we showed that the sensorimotor capabilities of people with schizophrenia directly influence their hand dexterity. Based on these findings, clinical practitioners should consider assessing the sensorimotor performance of the hands of patients with schizophrenia and develop effective programs for sensorimotor interventions in order to help these people to meet their occupational and daily needs. Moreover, the novel objective and quantitative assessments, the MTT and the PHUA, introducing in this study seem to be feasible for measuring the sensorimotor performance of patients with schizophrenia during clinical practice.

## Data Availability

The datasets analyzed during the current study are not publicly available due data protection requirements, but anonymous data or file could be available from the corresponding author on reasonable request.

## List of Abbreviations

9-HPT: Nine-Hole Peg Test
ADL: Activities of daily living
CGI-S: Clinical Global Impression-Severity
ESRS: Extrapyramidal Symptom Rating Scale
FDT: Functional Dexterity Test
FP_peak_: The peak pinch force during the lifting phase
FL_max_: The maximum load force at the onset of the maximum rate of upward acceleration
JTHFT: Jebsen-Taylor Hand Function Test
MMSE: Mini-Mental State Examination
MTT: Manual Tactile Test
PHUA: Pinch-holding-up-activity
PPT: Purdue Pegboard Test
SRG-PSP: Self-Reported Graphic version of the Personal and Social Performance
SWM: Semmes-Weinstein Monofilaments
VCWS 8: VALPAR Component Work Sample-8
