# Correction: Are the sensorimotor control capabilities of the hands the factors influencing hand function in people with schizophrenia?

**DOI:** 10.1186/s12888-024-05851-8

**Published:** 2024-06-04

**Authors:** Yu-Jen Lai, Yu-Chen Lin, Chieh-Hsiang Hsu, Huai-Hsuan Tseng, Chia-Ning Lee, Pai-Chuan Huang, Hsiu-Yun Hsu, Li-Chieh Kuo

**Affiliations:** 1 Department of Occupational Therapy, College of Medicine, National Cheng Kung University, No.1, University Road, Tainan City, 701 Taiwan; 2 Department of Occupational Therapy, Da-Yeh University, Changhua, Taiwan; 3 Department of Psychiatry, National Cheng Kung University Hospital, College of Medicine, National Cheng Kung University, Tainan, Taiwan; 4 Department of Physical Medicine and Rehabilitation, National Cheng Kung University Hospital, College of Medicine, National Cheng Kung University, No.1, University Road, Tainan City, 701 Taiwan; 5 Department of Biomedical Engineering, College of Engineering, National Cheng Kung University, Tainan, Taiwan; 6 Medical Device Innovation Center, National Cheng Kung University, Tainan, Taiwan

**Correction: BMC Psychiatry (2023) 23:807** 10.1186/s12888-023-05259-w

Following the publication of the original article [1], the authors identified that the IRB code in the Methods section was incorrect. The correct IRB code is given below.

The incorrect IRB code is: B-ER-107-084.

The correct IRB code is: B-ER-109-188.

The original article [1] has been corrected.
